# A time-use study of community health worker service activities in three rural districts of Tanzania (Rufiji, Ulanga and Kilombero)

**DOI:** 10.1186/s12913-016-1718-6

**Published:** 2016-09-01

**Authors:** Kassimu Tani, Allison Stone, Amon Exavery, Mustafa Njozi, Colin D. Baynes, James F. Phillips, Almamy Malick Kanté

**Affiliations:** 1 Ifakara Health Institute, P.O. Box 78373, Dar es Salaam, Tanzania; 2 Heilbrunn Department of Population and Family Health, Mailman School of Public Health, Columbia University, New York, USA

**Keywords:** Community health worker, Time use, Connect project, Tanzania

## Abstract

**Background:**

Despite expanding international commitment to community health worker (CHW) deployment, little is known about how such workers actually use their time. This paper investigates this issue for paid CHWs named “Community Health Agents,” which in Swahili is “*Wawezeshaji wa Afya ya Jamii”* (“*WAJA*”), trained for 9 months in primary health care service delivery and deployed to villages as subjects of a randomized trial of their impact on childhood survival in three rural districts of Tanzania.

**Methods:**

To capture information about time allocation, 30 *WAJA* were observed during conventional working hours by research assistants for 5 days each over a period of 4 weeks. Results were presented in term of percentage time allocation for direct client treatment, documentation activities, health education, health promotion non-work-related activities and personal activities.

**Results:**

During routine 8-h workdays, 59.5 % of *WAJA* time was spent on the provision of health services and other work-related activities. Overall, *WAJA* spent 27.8 % of their work on traveling from home to home, 33.1 % on health education, 9.9 % of health promotion and only 12.3 % on direct patient care. Other activities related to documentation (7.8 %) and supervision (2.5 %).

**Conclusions:**

Results reflect the pressing obligations of *WAJA* to engage in activities other than direct work responsibilities during routine work hours. Time spent on work activities is primarily used for health education, promotion, moving between households, and direct patient care. However, greater effort should be directed to strengthening supervisory systems and follow-up of challenges *WAJAs* facing in order to increase proportion of working hours.

## Background

Global commitment to primary health care development was accelerated by the 1978 Alma Ata International Conference on Primary Health Care (PHC) and expanded by the proliferation of community health workers (CHW) programs that has spanned three decades. Using a variety of strategies, CHWs have been deployed to implement a range of health service activities and act as agents to foster social change [1–3]. They are typically community members selected to perform functions related to basic preventive, curative, and promotional healthcare delivery [3, 4]. Most programs around the world rely upon CHWs who have minimal training and have no university-based degree training [5, 6]. In sub-Saharan African and other developing countries where CHWs are utilized, they receive brief technical training, usually focused on the provision of specialized packages, such as integrated packages (maternal and child health), focused package (antenatal care, newborn and family planning) and specific burden of diseases (Tuberculosis, malaria, HIV-AIDS, etc) [3, 7–9]. Their orientation and training range from 5 days to 9 months for programs where CHWs serve as multi-purpose paramedics [4, 10, 11].

Mounting evidence suggests that CHWs specializes on various health services have improved utilization of those services, for example the uptake of maternal and child health services by increasing community awareness about newborn care, prevention of communicable diseases and the use of rapid diagnostic tests to diagnose and treat malaria [6–8, 12]. Several important programs involve CHWs in the management of childhood illness [6]. However there are voluntary and salaried CHW. The voluntary dedicate a limited time of about 2 to 4 h a week differently from salaried, who are full time on targeted intervention [13]. The major factor limiting time devoted by voluntary and salaried CHWs to the community health works is poor remuneration, supervisions and supplies [14]. A number of countries have either scaled up CHW programs [4] or seek to address the shortage of health workers by expanding CHW coverage and care [14, 15].

Tanzania is one of the countries aiming to increase coverage of health services provision and improve the continuum of care between the community and health facilities through the engagement of CHWs. This is pointed out in a document named Tanzania’s Primary Health Care Improvement Policy of 2007 (known by its Swahili acronym, “MMAM”), which calls for the development of a paid official government cadre of CHWs and reinforced in the Health Sector Strategic Plan IV 2015–2020. With development of policy guides, and presence of different types of CHW in Tanzania, the Tanzania Ministry of Health and Social Welfare (MoHSW), the Ifakara Health Institute (IHI) and the Mailman School of Public health of the Columbia University (MSPH-CU), in collaboration with the Tanzania Training Centre for International Health (TTCIH) developed curriculum and trained a standard CHW for 9 month. The trained cadre of CHWs was called Community Health Agents (CHAs) which in Swahili is “*Wawezeshaji wa Afya ya Jamii”* (“*WAJA*”). *WAJAs* are working in three rural districts of Tanzania in randomized cluster trial intervention called Connect Project. The project aims to strengthen the health system by utilizing *WAJAs* whom they are connecting communities to the health system, with the aim of reducing maternal mortality and improving child health to achieve MDGs 4 and 5 [16].

*WAJAs* perform a number of activities including health education, health promotion, curative and non-health-related work activities such as documentation and preparation of routine reports. Understanding the proportion of time that *WAJAs* spend on each activity and implication for the amount of time devoted to direct patient care is a key in understanding and strengthening the implementation of the *WAJA* program. Previous study in Southern Tanzania had shown that a minority of facility health worker time is spent on direct patient care, in spite of its importance relative to other activities [17]. A study from Ethiopia found that health extension workers spent less time at community (37 %) compared to time spent at health post (51 %) [18]. Another study from rural Ghana showed that community health officers and health extension workers spent only 12% of their time for direct patient [19]. There is a need of exploring the time spent by CHW at community as there evidence that even at rural setting the health worker spent less than 1 % of their time in preventive and outreach services [20] that purposely has to be shared and performed by CHW.

The present study focused on understanding how the *WAJAs* spend their time on all tasks, grouped into community health services activities, other work-related activities and personal activities. In addition, the study assessed the relative proportion of observed CHW activities on maternal and child health services, health education, health promotion and documentation to better understand how the functioning of the *WAJA* program reflects the project’s focus on maternal and child health. This will point the time *WAJAs* spent at community in this project, and picture out what should be done during scaling up.

## Methods

### Study area and setting

The study took place in the Rufiji, Ulanga and Kilombero districts of Tanzania. In 2012, the Ifakara Health Institute in partnership with Rufiji, Ulanga and Kilombero districts deployed 142 full-time *WAJAs*, paid an amount equivalent to $120 USD per month, through their districts and deployed to some of the villages of these districts. *WAJAs* were attached to the existing health system and received supportive supervision from the clinicians based at the nearest health facility located within the village or near village. From the project setting the *WAJAs* received supervision from project coordinator, district CHW focal person and village supervisor [16].

### Participants and sampling

The *WAJAs* were selected from their respective villages, trained for 9 months and deployed back to their original villages. They were provided with the essential equipment to facilitate their daily activities, including a bicycle, a mobile phone, malaria rapid diagnostic tests (RDTs), behavior change communication materials, and medicines such as oral rehydration salts, ant-malarias, antibiotics to treat pneumonia, contraceptive pills, condoms etc. More information about the Connect intervention is provided in previous publication [16].

The *WAJAs* participated in this study were drawn from a random selection of 14 out of the 50 villages where *WAJAs* were deployed. The number of *WAJAs* in the sampled villages ranged from one to four, and all *WAJAs* in the selected villages were eligible for participation in the study. A total of 30 *WAJAs* were working in the 14 selected villages. Participation in the study was voluntarily and written consent was obtained from each participant before the onset of the study.

### Measurement tool

To capture information about time allocation direct observation was used along with a multidimensional work classification tool (Table 1) for sampling activities [20, 21]. This tool facilitates data recording during observation and can facilitate recording of all task to the most detailed. A number of studies have been done using such tools to look at similar issues in developing countries. Woelk and colleagues [22] looked at two urban clinics in Harare and Zimbabwe; Desai and McCaw [23] assessed time use among health workers at health facilities in Jamaica. In Ghana, Frimpong [19] assessed Community Health Officers working at the village level in rural Ghana and in Zambia, Counihan and colleague [8] assessed community health workers who uses RDTs to diagnose malaria. Each of these studies used activity sampling techniques and concluded that facility-based health workers could exercise flexibility in the time they allocated to different tasks. However, none of these studies were on CHWs working full time and tasked with providing multiple services entirely at the household level.

**Table 1:** Direct Observation Activity Classification Guide

Code	Activity
	1. Documentation and Reporting
10	Documentation (writing in register, patient books)
11	Completing monthly reports
12	Other documentation and reporting related activity
	2. Supervision and Training
21	Visit from Village Supervisor
22	Visit from Facility Supervisor
23	Visit from Intervention Coordinator
24	Visit from WAJA focal person
25	Phone call with Village Supervisor
26	Phone call with Facility Supervisor
27	Phone call with Intervention Coordinator
27	Phone call with WAJA focal person
29	Training from the project
210	Training from other stakeholders
211	Other supervision/training related activity
	3. Patient Care/Treatment
31	Family Planning provision
32	Deworming
33	Examination/Treatment of disease or injury (under 5)
34	Examination/Treatment of disease or injury (over 5)
35	Talking to patient, but non-health related
36	Travel with referred patient/patient related
37	Other patient care/treatment related activity
	4. Individual Patient Health Education and Counseling
41	Pregnant client - Healthy pregnancy
42	Pregnant client - Safe delivery
43	Pregnant client - Newborn care
44	Pregnant client - Breastfeeding
45	Pregnant client - Family Planning
46	Pregnant client - Other
47	Postnatal - Newborn care
48	Postnatal - Breastfeeding
49	Postnatal - Family Planning
410	Postnatal - Other
411	Immunizations/Vitamin A
412	Growth monitoring (consult weight for age chart)
413	Family Planning
414	Malaria (bednet use, symptoms, treatment)
415	U5 illness (diarrhea, pneumonia)
416	Deworming
417	Nutrition
418	HIV/AIDS related education
419	Clean environment, personal hygiene, and sanitation
420	Health insurance promotion
421	Other disease/Injury/treatment information
422	Talking to patient, but non-health related
	5. Group Health Education and Counseling
51	Healthy pregnancy
52	Safe delivery
53	Newborn Care
54	Breastfeeding
55	Immunizations/Vitamin A
56	Growth monitoring
57	Malaria (bednet use, symptoms, treatment)
58	U5 illness (diarrhea, pneumonia)
59	Family Planning
510	Deworming
511	Nutrition
512	HIV/AIDS related education
513	Clean environment, personal hygiene, and sanitation
514	Health insurance promotion
515	Other disease/Injury/treatment information
516	Talking to group but non-health related
	6. Cleaning/Organization
61	Cleaning of supply room/cupboard
62	Organizing supplies
63	Other sanitation/organization related activity
	7. Other Work-Related Activities
71	Travel or moving between households and supply room
72	Other work-related travel
73	Assisting facility outreach services
74	Bicycle repair
75	Individual work for drafting work plan/schedule
76	Discussion with other WAJAs to form work plan
77	Communication with other WAJA (in-person or by phone)
78	Communication with village health committee
79	Communication with staff from a program/project
710	Attending village council meeting
711	Attending village health committee meeting
712	Attending health facility governing committee meeting
	8. Non-Work Related Activities
81	Lunch or Break
82	Non-work related communication
83	WAJA closed/left for the day
84	Personal activities or travel (non-work related)

Observation of *WAJAs* was conducted according to Harvey’s description of analysis of time [21] which stipulates that observation of activities must be carried out for a sufficient length of time, and must be recorded, examined and coded along several dimensions. The instrument used to measure *WAJA* activities in this study was developed with input from the project intervention coordinators and trainers to determine the various activities conducted by *WAJAs* throughout their typical workdays. A pilot was performed for 2 days to test the applicability of the instrument in Rufiji district. After review of the tool, the teams carried out additional pilot in Rufiji and Kilombero districts to ensure research assistants were conversant with the final tool in each area.

The final classification tool used to observe *WAJAs* included 78 distinct activities, divided into eight categories that were documentation and reporting, supervision and training, patient care/treatment, individual health education, group health education, *WAJA* office cleaning and organization, other health work-related activities and lastly *WAJA* personal activity (Table 1).

### Data collector training and data collection

Seven full time research assistants were trained on the use of the observation tool for 1 week. Then a day pilot was organized to test the tool in villages that were not sampled for the study. The observers were equipped to resemble *WAJAs*, with a bag and bicycle, in order to avoid conspicuousness and maintain a routine community environment as possible. Observers were trained to identify and they were provided with a sheet with a list of all tasks that *WAJAs* performs and were instructed not to interrupt any conversations or consultations that the *WAJAs* were engaged in. They were instructed to only ask the *WAJAs* questions if they were uncertain how to characterize the activity they were engaged in, and to only do so when the *WAJA* was not attending clients.

*WAJAs* are full time employees in the village with approximate working hours from 8:00 AM to 4:00 PM from Monday through Friday. Each *WAJA* was observed for 5 days. Days of observation for an individual *WAJA* were distributed across a 30-day period and took place on various days of the week to capture a varied sample of *WAJA* activities. Observation of each *WAJA* was conducted by the same research assistant for each of the 5 days of observation. Research assistants were present to observe *WAJA* activities from 8:00 AM until up to 5:00 PM, and completed observation when the *WAJA* finished their work for the day. Observations of *WAJA* activities were recorded in 10 min intervals. Research assistants selected the activity in the observation tool, that was most applicable to the activity being performed by the *WAJA* at the beginning of each interval, and recorded the activity code against the time of the observation. Data collection was done between July and August 2013, which was the harvest and dry season.

All procedures used in this study adhere to the STROBE guideline for reporting observation studies [24].

### Analysis

Two research assistants entered the data into EpiData and then transferred the data into Stata 12 using Stata transfer. Stata 12 was used to clean data; analysis was done using Stata 12 and Microsoft Excel. Analysis involved tabulations of the different activities performed by the *WAJAs* during observation to arrive at average amounts of time spent on each activity across the standard work day. Results were presented in term of figures and tables.

## Results

The attribute and pattern of *WAJA* working in the area shows that they are secondary school graduates. They are permanent residency of the villages were working. WAJA observed to be dedicated to their work all days of the week, with planned schedule from Monday to Friday. Six WAJA declared to attend a number of cases after 4.00 PM and over the weekend at their houses and even at mid night in case of emergencies.

**Fig. 1:**
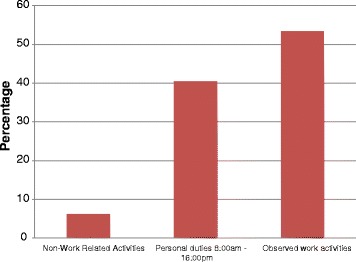
Mean proportion of working time over the hours from 8:00 AM – 4:00 PM

About 93.3 % (28 out of 30) of sampled *WAJAs* agreed to participate in the study and were observed. The 2 *WAJAs* missed were on maternal and annual leave. During work hours, *WAJAs* spent an average of 59.5 % of their time on community health service activities that is health activities (53.4 %) (Treatment, health education, health promotion and documentation) and related community work activities (6.1 %) (socialization and repair bicycle). The socialization is related to greetings and talking with members of the household before any health activity and also to *WAJA* participation to overall village ceremonies (funerals for example). The remaining 40.5 % of *WAJA* time activity are non-work-related activities such as personal errands, farming, or taking care of their families (Fig. 1).

**Fig. 2:**
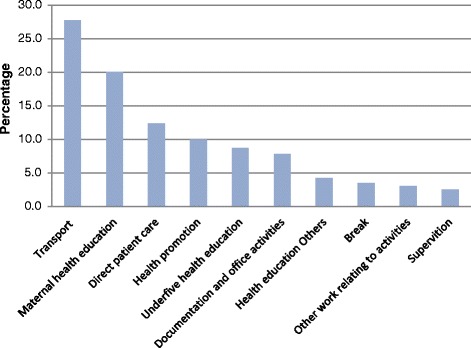
WAJA time use breakdown, 8.00 AM–4.00 PM to each category of community 416 health service work

The Fig. 2 excludes the personal activities done within the working time boundary. The large portion of time allocation of *WAJA* time was spent traveling from one household to another about 27.8 %. *WAJAs* allocated the most time on maternal health education (20.0 %), under-five health education (8.7 %), health promotion (9.9 %), and documentation and office activities (7.8 %). Other health-related work activities observed including supervision (2.5 %), health education not focused on maternal and child health topics (4.3) and break (3.5 %). In overall, *WAJAs* spent 42.9 % of their time on health education and promotion while the direct patient care was devoted to only 12.3 % of the total working duration (Fig. 2).

**Fig. 3:**
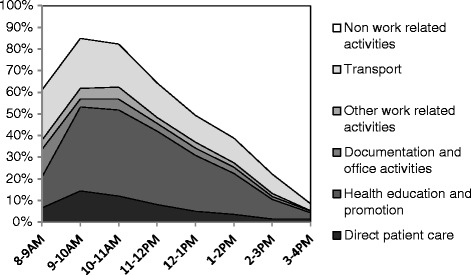
Mean hourly distribution of time allocation by activity per day

Figure 3 presents the average distribution of time allocation for each hourly period of the standard working day. Most of *WAJA* health service activities, including maternal health education, under-five health education and health promotion, were concentrated between 9:00 AM and 12:00 PM. From 8:00 AM to 9:00 AM, *WAJAs* spent an average of 60 % of their time providing health services. This increases to about 85 % between 9:00 AM and 11:00 AM, and then declines over the afternoon to less than 10 % by 4:00 PM. Personal activities like taking care of family and farming occurred mainly at the beginning of the day and after 2:00 PM. From 3 PM, *WAJAs* spent 90 % of their time on personal activities.

## Discussion

This study is one of the few studies to examine time use among CHWs and the first for those professionalized and salaried in Tanzania [16]. The finding that *WAJAs* are engaged in work-related activities for only 59 % of their working hours may be partly attributed to the fact that *WAJAs* are a new cadre in the Tanzanian health system and therefore require extensive supportive supervision and follow up [25] to ensure productivity. Because *WAJAs* are new to these responsibilities, they are in need of an enabling environment and quality support from the system [25, 26]. Typically, CHWs work in specific programs for an abbreviated period of time [14, 27] rather than as full-time, paid employees appeared to use few hours. Future implementation and scale-up of the *WAJA* program requires an understanding of the factors constraining the proportion of time that *WAJA* spend on community health services in the context of their full-time employment.

Findings showed that large portion of time been devoted is on promotion and preventive cases has also been observed by other study in Ethiopia [18]. The communities are surrounded by a number of health issues that need daily clarification and reminds. These are issues like maternal education, under five health education and other health promotion of which CHWs invest more time on them [18] compared to facility based staff even working in rural setting [20].

Our study found that traveling from one household to another appear to take a large portion of *WAJA* time similar to that of Odendaal in South Africa [28] and may be related to the dispersed settlement patterns of the study communities in which the *WAJAs* provide services at the doorstep level. Supervision appeared small as this cadre was designed to work independently in a community for the large part of the time. Similar study done in Ghana documented that, less frequently of supervision reduces the volume of care by health workers working alone even at health facilities [19]. Supervision as one of human resource management component is important as it creates a chance for discussion on challenging areas when services delivery.

## Limitations

This study is based on a relatively small number of participants and estimates of their working time are subject to a number of definitions and boundaries. In this case, boundaries have been influenced by the working location and preset working hours from 8:00 AM to 4:00 PM. Our study was unable to address the fact that *WAJAs* sometimes work outside the normal range of working hours, and therefore did not measure their activities off-hours such as response to emergencies, emergency provision of first aid and advice.

Another limitation is the fact that the study took place within a 30-day period, and therefore seasonal variations could not be observed. This study was conducted just after the end of the harvest season in Rufiji district and during the harvest season in Kilombero and Ulanga districts. Patterns of illness and *WAJAs* responses were therefore only representative of this small portion of the year. *WAJA* activities during the observed harvest and post-harvest seasons may have important differences compared to the rainy seasons when cultivation is taking place, because at this time, community members often reside on farms far away from their permanent homes. Understanding variation in *WAJA* time use in response to seasonal factors is important in this setting where the majority of community members are subsistence farmers.

Finally, the direct observation methods employed by this study are limited, that is *WAJAs* may have modified their work routines in response to the presence of the research assistants observing their work. Rather than capturing a sampling of actual *WAJA* work routines, direct observation rather captures a sampling of *WAJA* work routines in the presence of a research assistant given the task of observing their work. While this method may not capture a completely accurate portrayal of *WAJA* work routines, direct observation is considered more reliable than *WAJA* self-reports of their time use [24].

## Conclusion

*WAJA* are a new cadre playing a crucial role in linking communities to the health system. This study observed that *WAJAs* spent a large portion of their time as intended in health education and promotion. However, greater effort should be directed to strengthening supervisory systems and follow-up of challenges *WAJAs* facing in order to increase the proportion of working hours spent on provision of services to their clients each day. In addition, it will be important to identify factors that facilitate and or hinder *WAJA* activities seasonally, as they work in rural area where most clients are subsistence farmers who move to their farms.
